# Study on the release and sensory perception of encapsulated d‐limonene flavor in crystal rock candy using the time–intensity analysis and HS‐GC/MS spectrometry

**DOI:** 10.1002/fsn3.1372

**Published:** 2020-01-09

**Authors:** Shahrzad Vatankhah Lotfabadi, Seyyed Ali Mortazavi, Samira Yeganehzad

**Affiliations:** ^1^ International Campus Ferdowsi University of Mashhad Mashhad Iran; ^2^ Department of Food Science and Technology Faculty of Agriculture Ferdowsi University of Mashhad Mashhad Iran; ^3^ Department of Food Processing Research Institute of Food Science and Technology Mashhad Iran

**Keywords:** flavor release, flavored rock candy, matrix, serving temperature, time–intensity

## Abstract

This research was conducted to evaluate encapsulated d‐limonene perception and release in rock candy. Microcapsules with wall materials of 75/25 of gum Arabic/Maltodextrin by 20% of wall materials) were produced for using in rock candy. To evaluate the flavor release from rock candy by time–intensity method, a model system was developed and time–intensity sensory evaluation was conducted by trained sensory panelists in order to determine the effect of three different matrices (water, water and flavored rock candy, and water with flavored rock candy and citric acid (pH = 3) at three serving temperatures (10, 45, and 75°C) on the perception of d‐limonene release. Results showed that release of d‐limonene from flavored rock candy with acid citric (pH = 3) at 75°C had the highest perceived sensation whereas the matrix of microcapsule in water at 10°C had the lowest perception. On the other hand, increasing the temperature from 10 to 75°C had significant effects on the release and perception of d‐limonene (*p* < .05). Headspace gas chromatography–mass spectrophotometry confirmed results from time–intensity sensory evaluation, which indicated that the release of d‐limonene increased in the presence of sucrose and citric acid (pH = 3).

## Graphical abstract

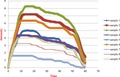

**Highlights**

- The present study considered time–intensity as a dynamic sensory evaluation method to evaluate perception of encapsulated d‐limonene in flavored rock candy.
- In this research, a model system containing sucrose and citric acid was developed to better perception of microencapsulated d‐limonene release.
- Headspace GC/MS was applied to scrutinize the release of encapsulated d‐limonene in different matrices and temperatures.
- Higher temperature was shown to be more effective on perception and release of d‐limonene.

## 1 INTRODUCTION

**Figure 1:**
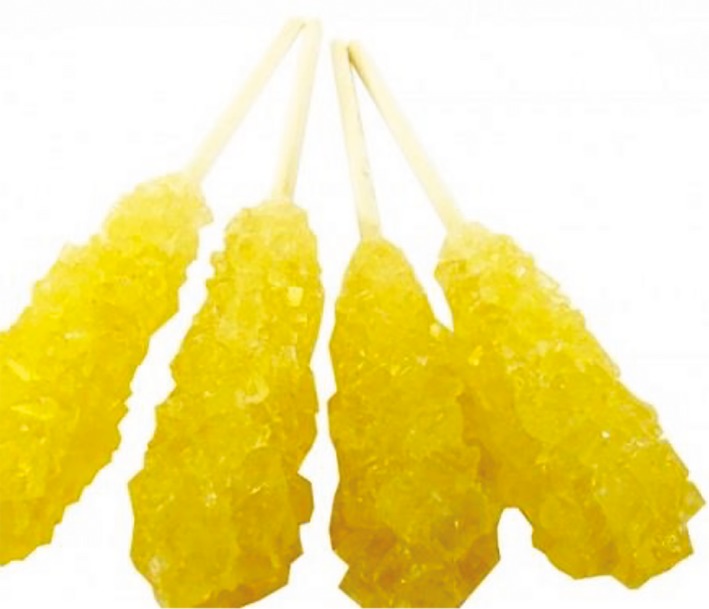
Stick Rock candies

Rock candy is a very unique sugar product originating from India and Persia, where it is called Mishri and Nabat, respectively (Gholamhosseinpour, Varidi, Elahi, & Shahidi, 2008). It is produced by supersaturation of sucrose solution followed by cooling in order to crystalize (Van der Poel & Schiweck, 1998) and has both sweetening and medical applications. Rock candy is usually consumed with tea, soft drinks, and alcoholic beverages. Although its production is time‐consuming, crystal rock candy is simply composed of large crystals of sucrose. These crystals are either suspended from string, attached to a stick or left loose (Hartel, 2001; Figure 1). Due to the growing demand for this confection, manufacturers attempt to diversify products to satisfy consumers more. Production of flavored rock candy and meanwhile preventing undesired changes during processing is challenging. Encapsulation technique seems to be an effective method for protecting flavoring agent overprocessing and preserving the final product.

Microencapsulation is the packaging of small particles, which make up a core, with a film of continuous polymer. Flavoring agents are often sensitive to oxygen, heat, light or acid, and can be preserved through microencapsulation, which also allows a refcontrolled release of the core contents (Krishnan, Kshirsagar, & Singhal, 2005). This resultant increase in flavoring agent chemical stability is of great importance in the food industry, where hydrophobic flavors are incorporated into powders by encapsulation (Yoshii et al., 2001). The way in which these flavors are released, including how they can be released under control in foods, comprises an important aspect in the estimation of the potential storage period (Pszczola, 1998; Reineccius, 1995; Whorton, 1995).

d‐limonene is a major component of various citrus oils including lemon, lime and orange, and has a lemon‐like smell which is commonly consumed with wide range of soft and alcoholic drinks. It is classified as a monocyclic monoterpene and emits a pleasing citrus scent. Being both a fragrant and a flavoring agent, d‐limonene has widespread usage in soaps, foods, perfumes, chewing gum, and beverages (Li & Chiang, 2012). Over the production process of rock candy, microencapsulation of d‐limonene ensures preservation against heat and other destructive factors.

A number of studies have shown the effects of tastants on perceived flavor intensity and most of them suggest an enhancement of flavor perception by sweetness and sourness (Bonnans & Noble, 1993; McBride & Johnson, 1987; Pfeiffer, Hort, Hollowood, & Taylor, 2006). Interactions occur both within and between modalities. An example of the former is how sugar subdues the sour taste of citric acid (Curtis, Stevens, & Lawless, 1984), and two examples of the latter are the impact of aroma on the perception of flavor (Pfeiffer et al., 2006).

Throughout the consumption of food, sensory perception changes due to its dynamic nature (Cliff & Heymann, 1993). Processes such as breathing, salivation, chewing, tongue movement, and swallowing impact the sensory perception of food, moment by moment (Lawless & Heymann, 2010). In the past, conventional methods of measuring sensation were based on a static perception of food for a specific moment. However, methods to capture moment by moment sensory perception by employing dynamic techniques have been developed and are much closer to reality (Dijksterhuis & Piggott, 2000). So as a new product which contains d‐limonene microcapsule in its structure, investigation of the time and place of interactions must be identified as they can affect how flavor is perceived, in addition to other characteristics, thereby modifying the products’ sensory profile (Hewson, Hollowood, Chandra, & Hort, 2008). Such interactions may take place at different levels, including physical interactions between constituents which lead to fluctuations in volatile release (Da Porto, Cordaro, & Marcassa, 2006).

Time–intensity (TI) sensory analysis has been widely utilized for many years for products including sweeteners (Melo, Bolini, & Efraim., 2009; Mosca, Velde, Bult, Boekel, & Stieger, 2010; Ujikawa & Bolini, 2004) and beverages (Rodrigues, Andrade, Bastos, Coelho, & Pinheiro, 2016; Sokolowsky & Fischer, 2012; Sokolowsky, Rosenberger, & Fischer, 2015; Valentova, Skrovánková, Panovská, & Pokorný, 2002; Zorn, Alcaire, Vidal, Giménez, & Ares, 2014). This technique is directed toward giving panelists the ability to report how taste is perceived at different moments. As panelists report their perceptions moment by moment, scientists can quantify changes in a specific property over time. The resulting parameters for each sample are the maximum perceived intensity (Imax), take taken to reach maximum intensity (Tmax), the shapes and rates of the increase to Imax, the decrease to half of Imax and the decrease to the point of extinction, the area beneath the curve (AUC), and the total sensory duration (DUR; Lawless & Heymann, 2010).

Sugars are able to affect how flavor is released from some flavoring agents, altering their volatility; furthermore, the effect of a change in pH on flavoring agent release has also been investigated (Friel, Linforth, & Taylor, 2000; Hansson, Andersson, & LeufveÂn, 2001a). The aim of the present study were (a) to study d‐limonene release from crystal rock candy, (b) to develop a model system to study flavor release from encapsulated molecules, and (c) to develop time–intensity method to evaluate flavor perception from a novel confectionery product.

## 2 MATERIALS AND METHOD

### 2.1 Materials

d‐limonene (ρ = 0.84g/cm^3^ at 20°C, Molar Mass: 136.24 g/mol) was purchased from Nacalai Tesque. Gum Arabic and Maltodextrin (DE = 16.5–19.5) were supplied from SDFCL and Sigma‐Aldrich, respectively. Sugar was prepared from Paniz Shahd Binalood Co. Citric acid was obtained from Sigma‐Aldrich Chemistry. Distilled water was used for preparing all solutions. All organic chemicals used in the analyses were of the analytical grade.

### 2.2 Preparation of flavored rock candy

The production of rock candy was performed by using sugar and water; first, water was heated up to 100°C, and then sugar was gradually added up to a level of total solution by constant agitating; Heating would be continue until the brix of solution reach to 83; then, microcapsule (d‐limonene was encapsulated with mixture of gum Arabic and maltodextrin at ratio of 3:1 by spray drying; total solid content of wall materials was 20% w/w; Vatankhah Lotfabadi, Mortazavi, Yeganehzad, & Sadeghian, 2018) by amount of 0.04 g for each rock candy added to the syrup and then flavored syrup was incubated so that the crystallization occurred over 16 hr inside the oven by gradual descending temperature from 80 to 40°C. The final product has 10 g weights for following sensory evaluation.

### 2.3 Sensory analysis

#### 2.3.1 Training session

In this research, time–intensity method was developed to evaluate flavor release from rock candy; therefore, to better perform the test, a model system was developed (as described in 2.3.2). Five 1‐hr sessions were conducted in order to train and acquaint panelists with the time–intensity methodology in developed model system. For data acquisition and data analysis, the Sensomaker program (Kiumarsi et al., 2019; Nunes & Pinheiro, 2012) was used. Through a graphical interface in the form of 10‐point scale, with 0 meaning no perception and 10 signifying an extreme perception of d‐limonene, each panelist indicated the intensity of the attribute of sample with monadic presentation, using complete block design (Wakeling & Macfie, 1995).

#### 2.3.2 Flavor perception using time–intensity (TI) analysis

The sensory panel consisted of ten trained judges (five males and five females; age ranging 26–40 years) from the Research and Development Department of Saharkhiz Company and Research Institute of Food Science and Technology (RIFST), who were all skilled in food and beverage sensory evaluation. The attribute evaluated was d‐limonene flavor, and the samples were presented in a monadic way, using a balanced complete block design (Wakeling & MacFie, 1995).

#### 2.3.3 Experimental procedure

The nine samples comprised d‐limonene microcapsule in water at each of 10, 45, and 75°C, flavored rock candy in water at each of 10, 45, and 75°C, and flavored rock candy in water and citric acid at each of 10, 45, and 75°C. All samples were labeled with randomized three‐digit codes and were presented in 30 ml proportions of solutions; sensory evaluation was conducted in an air‐conditioned room (20°C). The panelists began evaluating by clicking on a “start” button and consumed 10 ml of sample (a gulp of the sample over 2 s), during the next 60 s, the panelists began evaluating by clicking on a “start” button and consumed a gulp of the sample over 2 s. During the next 20 s, using the mouse, the panelists indicated the perceived intensity of d‐limonene on the scale (Table 1). At the end of the analysis, the evaluation stopped after the panelists reached the left end of the scale after 60 s. a message indicating the end of the test appeared and the panelists rinse their mouths with mineral water in order to prepare for the next sample. All samples were presented in randomized order and two replications.

**Table 1:** Sample preparation

Samples	Preparation
d‐limonene microcapsule in water (10°C)	Encapsulated d‐limonene dissolve in water
d‐limonene microcapsule in water (45°C)	Encapsulated d‐limonene dissolve in water
d‐limonene microcapsule in water (75°C)	Encapsulated d‐limonene dissolve in water
Flavored rock candy in water (10°C)	Flavored rock candy by d‐limonene microcapsule dissolve in water
Flavored rock candy in water (45°C)	Flavored rock candy by d‐limonene microcapsule dissolve in water
Flavored rock candy in water (75°C)	Flavored rock candy by d‐limonene microcapsule dissolve in water
Flavored rock candy in water and citric acid (10°C)	Flavored rock candy by d‐limonene microcapsule dissolve in water with citric acid (pH = 3)
Flavored rock candy in water and citric acid (45°C)	Flavored rock candy by d‐limonene microcapsule dissolve in water with citric acid (pH = 3)
Flavored rock candy in water and citric acid (75°C)	Flavored rock candy by d‐limonene microcapsule dissolve in water with citric acid (pH = 3)

The time–intensity course was characterized by time when intensity is 90% of Imax at increasing part of the curve, area under the curve, and plateau which indicates on time interval which the intensity is ≥90% of Imax.

### 2.4 Headspace gas chromatography–mass spectrometry

Static headspace analysis followed by gas chromatography–mass spectrometry (GC‐MS) analyses was done on flavored rock candy at 10°C, 45°C, and 75°C.

All analyses were carried out in duplicates; samples were prepared in 30‐ml volumetric flasks with valve caps; After completely dissolve crystal rock candy, 1,000 µl of the vapor phase from each sample flask at each temperature was injected into GC‐Agilent Technologies model: 7890A and MS‐Agilent Technologies model: 5975c equipped with a HP‐5MS 5% phenyl methyl siloxane capillary column (325°C: 30 m × 250 µm × 0.25 µm) with helium flow rate of 1 ml/min. The column was held at 50°C for 3 min and then programmed to 270°C at 20°C/min.

### 2.5 Statistical analysis

An analysis of variance (ANOVA) was applied for the scores of each panelist and for selected parameters consisting of maximum intensity (Imax), running time of maximum intensity (plateau), and area under the curve (area); the probability level was *p* = .95. Tukey's test was applied to compare the averages of samples using the Sensomaker software (Pinheiro, Nunes, & Vietoris, 2013). The data of mean TI curves were presented in graphic form by using the Microsoft Excel 2012.

## 3 RESULTS AND DISCUSSION

### 3.1 Effect of matrix and temperature on d‐limonene perception

**Figure 2:**
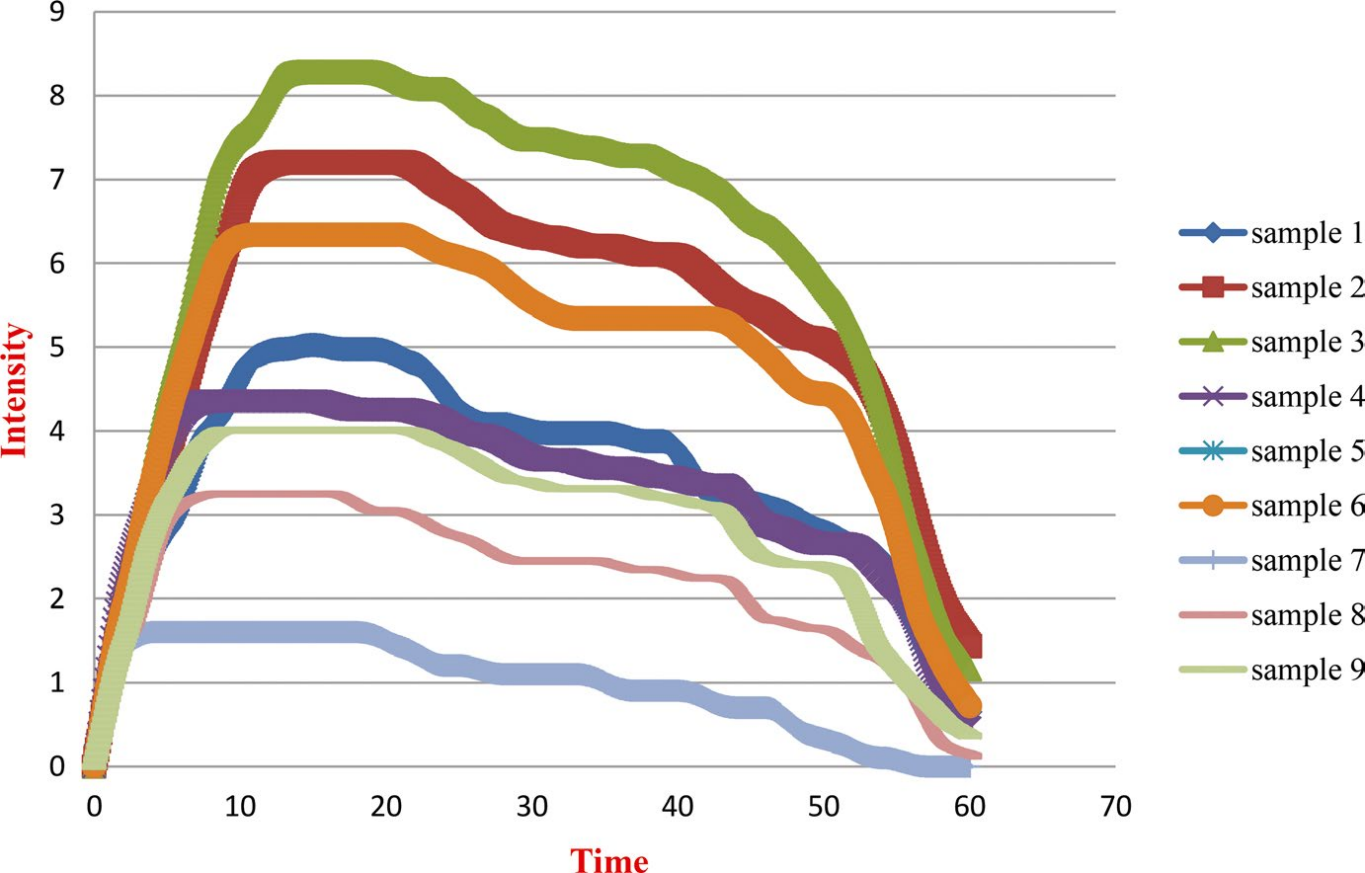
Time–intensity curves obtained for d‐limonene intensity over time (10 s) for d‐limonene microcapsule in water (75°C) as sample 1, flavored rock candy in water (75°C) as sample 2, flavored rock candy in water and citric acid (75°C) as sample 3, d‐limonene microcapsule in water (45°C) as sample 4, flavored rock candy in water (45°C) as sample 5, flavored rock candy in water and citric acid (45°C) as sample 6, d‐limonene microcapsule in water (10°C) as sample 7, flavored rock candy in water (10°C) as sample 8, flavored rock candy in water and citric acid (10°C) as sample 9

The effect of sucrose, citric acid, and water as blank sample on the release of d‐limonene was investigated at three serving temperatures which are usual temperatures for consumption of tea and cold drinks; intensity of d‐limonene flavor was the only attribute analyzed by assessors, and time–intensity curves for d‐limonene microcapsule in three matrixes and three temperatures (10, 45, and 75°C) are presented in Figure 2.

Table 2 shows the analysis of variance (ANOVA) and Tukey's mean test. Figure 2 shows d‐limonene intensity over time for samples with different temperatures and matrices. As observed in Figure 2, all of the samples had the same release trends and time–intensity profile for d‐limonene but differed in relation to their temporal profiles which are presented in Table 2.

**Table 2:** Means of time–intensity parameters for evaluated lemonades

Sample	Definition	*I*_max_	Plateau	Area
1	D‐limonene microcapsule in water (75°C)	5.08^de^	20.42^ab^	211.72^c^
2	Flavored rock candy in water (75°C)	7.20^g^	28.54^ab^	328.55^e^
3	Flavored rock candy in water and citric acid (75°C)	8.28^h^	19.1^b^	377.55^f^
4	D‐limonene microcapsule in water (45°C)	4.39^cd^	22.72^ab^	201.98^c^
5	Flavored rock candy in water (45°C)	5.81^ef^	20.59^ab^	268.92^d^
6	Flavored rock candy in water and citric acid (45°C)	6.34^f^	19.64^ab^	292.66^de^
7	D‐limonene microcapsule in water (10°C)	1.75^a^	17.17^a^	69.18^a^
8	Flavored rock candy in water (10°C)	3.48^b^	19.93^ab^	149.43^b^
9	Flavored rock candy in water and citric acid (10°C)	4.13^bc^	24.63^ab^	185.14^bc^

Note

Means followed by the same letter in the column did not differ significantly (*p* ≤ .05) each other by Tukey test. Imax—Maximum intensity, Plateau—running time of maximum intensity, Area—area under the curve.

Analysis of variance on samples reveals significant differences among parameters including maximum intensity and area under the curve. As shown in Table 2, we found that the maximum intensity of d‐limonene varied significantly (*p* < .05) among samples, sample 3 (Imax = 8.28), sample 2 (Imax = 7.20), and sample 6 (Imax = 6.34) put in order from first to last, respectively. Also, sample 7 had the lowest Imax (1.75). Furthermore, a similar trend was obtained for the area under the curve. However, regarding the plateau parameter, which indicates the duration of d‐limonene flavor, there were no significant differences among them. The results thereby showed that across all the evaluated samples, sugar, citric acid, and higher temperature had a significant effect on d‐limonene perception intensity.

Investigating the area under the curve, the samples’ trend of time intensity was observed (Table 2). Sample 3 and sample 2 showed a more prolonged time for release of d‐limonene and a greater maximum intensity of the stimulus. This resulted in the greatest area under the curve for both cases such that sample 3 and sample 2 displayed the highest area by means of 377.55 and 328.55, respectively. Moreover, the lowest area was associated with sample 7 with a mean of 69.18 (*p* < .05). Regarding the plateau, apart from significant difference between sample 3 and 7, other samples showed no significant differences (*p* > .05) during the time interval for maximum intensity. The results indicating that citric acid (pH = 3) had a pronounced effect on the intensity of d‐limonene sensory perception. Furthermore, the role of temperature on intensification of d‐limonene flavor was proven by the panelists’ perception.

The previous studies have suggested (Frank & Byram, 1988; Schifferstein, 1995) the compatibility of flavor‐tastant pairing had a significant effect on predicting influences on perception. By immersing rock candy to the water solutions, an increase in the d‐limonene release in gas phase was extensively observed (*p* < .05). This probably happened as a reaction to the “salting out” effect of sucrose (Voilley, Simatos, & Loncin, 1977) by which sucrose interacts with water, causing an increase in the concentration of flavor compounds in the remaining “free water” (Voilley et al., 1977). The “salting out” effect of glucose or sucrose leads to the release of aroma compounds coming from sugar solutions (Friel et al., 2000; Hansson et al., 2001a; Hansson, Andersson, LeufveÂn, & Pehrson, 2001b; Nahon, Koren, Roozen, & Posthumus, 1998; Voilley et al., 1977). Interactions of sugars and water causes the increase of the concentration of aroma compounds in the vapor phase (Lubbers & Guichard, 2003). The concentration of carbohydrate directly affects the viscosity of the system, and it also affects the retention and release of flavor compounds (Naknean & Meenune, 2010). As shown by the results, at any specifically determined temperature, by adding sucrose in a form of rock candy in the media, a more pronounced release of d‐limonene was significantly observed (*p* < .05). This was probably due to the “salting out” effect of sucrose (Voilley et al., 1977).

As Log *p*‐values can be used to define the hydrophobicity of a flavor compound, negative log *p*‐values can indicate a hydrophobic compound. The release of d‐limonene was high due to its strong nonpolar nature (Naknean & Meenune, 2010).

A decrease in the diffusion coefficients of aromatic compounds has been observed with increasing sugar content of the solution (sucrose, glucose), by which the viscosity increases.

When flavored rock candy was added to the matrix containing citric acid (pH = 3), d‐limonene release was significantly increased in headspace concentration compared to the other samples.

Samples which containing citric acid showed dramatic increases in the release of d‐limonene both in headspace and TI analysis in comparison with samples without citric acid, which confirms the increase of d‐limonene release as a result of the presence of citric acid. Citric acid is a triprotic acid, and according to calculations, the first form of dissociation is the dominating form in this system. This dissociated form might have a greater tendency than the nondissociated citric acid to interact with chemical compounds (Lindsay, 1985). Changes in pH might also affect the flavor compounds themselves. Increasing the pH will shift the equilibrium so that larger amounts of the citric acid are in the dissociated form, whereas lowering the pH leads to a larger amount of the nondissociated form (Bennett, 1992).

A number of studies have presented various effects of tastants on perceived flavor intensity and mostly express an enhancement of flavor perception by sweetness and sourness (Bonnans & Noble, 1993; McBride & Johnson, 1987; Pfeiffer et al., 2006), but this may depends on the congruency or in other words, the predicted proportionality of the taste–aroma pairing (Dalton, Doolittle, Nagata, & Breslin, 2000; Diamond, Breslin, Doolittle, Nagata, & Dalton, 2005; Frank & Byram, 1988; Schifferstein, 1995).

Hydration causes a change in the amount of “free water,” and the presence of solutes within a solution has been shown to affect the partition coefficient of the volatiles, their molar concentration, and their activity coefficient (Friel et al., 2000; Hansson et al., 2001a; Lethanh, Thibeaudeau, Thibaut, & Voilley, 1992; Nahon, Harrison, & Roozen, 2000; Taylor, 1998; Voilley et al. (1977). Several scientists have shown that interactions between solutes and volatiles are dependent upon the nature of the volatile substance causing the headspace volatile concentrations to decrease, increase, or remain the same. They have suggested that any differences in perceived flavor intensity between tastants containing samples are not a result of alterations in the physical release of a volatile from the beverage matrix (Da Porto et al., 2006; Ebeler, Pangborn, & Jennings, 1988; Nahon et al., 2000, 1998).

Hewson et al. (2008) showed that the addition of tastants, namely acids and sugars, resulted in a total decrease in headspace volatile concentration in comparison with the samples with just volatiles in water. Friel et al., 2000; Rabe, Krings, & Berger, 2003 have claimed that solute concentration determines the status of volatile release profiles.

Hewson et al. (2008) also suggested that the addition of acid and sugar leads to an increase in perceived flavor intensity. They found greater perception of flavor intensity in samples to which tastants (glucose, fructose, citric acid, or lactic acid) were added in singular or combined forms. As this increase in perception cannot be identified as a result of alterations in volatile release, this result provides evidence for the presence of multimodal (taste–aroma) interactions within this system.

Taste–aroma enhancement appears to be mutual if the pairing is congruent. “Sweet‐smelling” aromas increase the perceived sweetness and suppress sourness (Djordjevic, Zatorre, Petrides, & Jones‐Gotman, 2004b; Frank, Ducheny, & Mize, 1989) and a limited effect can be seen with imagined odors (Djordjevic, Zatorre, & Jones‐Gotman, 2004a).

Hewson et al. (2008) claimed that the enhancement of citrus flavor intensity observed when adding both citric and lactic acid, and hypothesized that perceived sourness intensity would be enhanced by increasing citrus aroma.

The fact that the addition of acid would have various effects on the sweetness of a sugar solution was not totally unexpected; previous findings have shown suppression (Pangborn, 1961), little or no effect (Curtis et al., 1984; McBride & Johnson, 1987), or enhancement of sweetness by acids (Kamen, Kroll, Gutman, & Pilgrim, 1961).

As Horn (1981) stated on evaluating sweetness and several factors influencing its perception, it was suggested that sweetness of sucrose can somehow be suppressed by acidic ingredients such as citric acid.

The temperature of food plays a critical role in the release of food volatiles (Cardello & Maller, 1982; Moskowitz, 1973; Ventanas, Puolanne, & Tuorila, 2010b; Zellner, Stewert, & Rozin, 1988). Product temperature was shown to largely affect the sensory characteristics. Oral temperature also affected sensory attributes, but to a lesser extent. This suggests that the physical/chemical characteristics are dominating in stimulating sensations of flavor and texture properties, and that these characteristics are readily altered by a change in temperature (Engelen et al., 2003).

The various treatments had variant effects on the volatile release amounts that appear in the headspace, and thus, the samples may be perceived diversely during consumption (Patana‐anake & Barringer, 2015). Various temperatures were tested to evaluate different conditions for product consumption. Patana‐anake and Barringer (2015) suggested that temperature had a proportional effect on volatile release, meaning that an increase in temperature would cause an increase in volatile release levels and vice versa. As for the case of tomato juice, we observed the same trend.

Jouquand, Ducruet, and Giampaoli (2004) demonstrated that the air/water partition coefficient of volatile compounds in the headspace rises with increasing temperatures, thereby achieving greater volatile compound concentration in the headspace and resulting in enhanced flavor intensity.

Ventanas, Mustonen, Puolanne, and Tuorila (2010a) assessed the effect of increasing temperature on flavor perception in aromatic foods and found that increasing the temperature led to enhanced perceived flavor intensity. Also, Kähkönen, Tuorila, and Hyyönen (1995) and Ryynänen, Tuorila, and Hyvönen (2001) have stated that the odor intensity of cheese soup was stronger at 63°C than at 33°C, and the odor and flavor intensity of carrot, meat patty, and mashed potato increased as their serving temperatures increased from 25 to 65°C.

Sensitivity to NaCl was significantly higher in solution temperatures of 22°C and 37°C than at 0 or 55°C (Pangborn, Chrisp, & Bertolero, 1970). The perceived sweetness of sucrose solutions of low concentrations was reported to vary in direct proportion with solution temperature (Bartoshuk, Rennert, Rodin, & Stevens, 1982; Calvino, 1986; Green & Frankmann, 1987), where the sweetness was greater at higher temperatures.

Hansson et al. (2001a), Hansson et al. (2001b) showed that an increase in the concentration of sucrose (from 20% to 60% w/w) resulted in a significant increase (*p* < .05) in the release of isopenthyl acetate, ethyl hexanoate, cis‐3‐hexenyl acetate, linalool, and L‐menthone to the gas phase above the soft drink.

Ventanas et al. (2010a) claimed that serving temperature is an important factor affecting both acceptability and intensity of odor and flavor properties. The effect of serving temperature on flavor perception depends on the food and the sensory attributes evaluated.

Ryynänen et al. (2001) reported an increase in both the odor and flavor intensities with increased serving temperature in different meals components, including meat patties.

Lubbers and Guichard (2003) also confirmed by equilibrium headspace analysis when investigating the effects of glucose and corn syrups on flavor release from a fruit pastille model system. Significant differences were observed among the flavor intensities of gels produced with the different mixture includes sucrose and glucose, sucrose plus glucose and corn syrup (DE40), sucrose plus glucose and corn syrup (DE60) which the amount of sucrose was the same in all samples.

Hansson Andersson, and LeufveÂn (2001a), Hansson et al. (2001b) studied the effect of changes in pH on the release of flavor compounds from a soft drink‐related model system and expressed that headspace concentration of the esters and limonene was increased by adding citric acid in small amounts. High concentrations of the acids decreased the release of esters, likely due to the presence of the dissociated form of the acids.

Results also showed that the same amounts of added citric acid had no effect on flavor release when pH was moderated by sodium hydroxide.

The result of headspace GC‐MS is shown in Table 3 which indicate the release of d‐limonene among three matrices as well as three various temperatures were significantly different. It is clear that temperature had a significant effect on the release of d‐limonene, as proven by time–intensity. d‐limonene release from flavored rock candy in water and citric acid (75°C) was much higher that the release from other matrices; moreover, flavored rock candy in water and d‐limonene microcapsule in water stand in second and third places. These results are therefore in agreement with sensory analysis; and as Godshall (1997) claimed, different sugars (sucrose, invert sugar, and glucose syrup) interact with water to different degrees, increasing various water activity values, and therefore impacting the release of flavor compounds.

**Table 3:** Headspace GC/MS chromatogram of d‐limonene analysis

Sample temperature	Samples	Retention time	Pick Area
75°C	d‐limonene microcapsule in water	7.03	294352233
	Flavored rock candy in water	7.03	323968954
	Flavored rock candy in water and citric acid	7.029	342878045
45°C	d‐limonene microcapsule in water	7.018	135035659
	Flavored rock candy in water	7.022	171174857
	Flavored rock candy in water and citric acid	7.023	193472919
10°C	d‐limonene microcapsule in water	7.013	1100454459
	Flavored rock candy in water	7.016	134564007
	Flavored rock candy in water and citric acid	7.015	137768709

## 4 CONCLUSION

In this study, flavor release from crystal rock candy was evaluated by developing a model system. Effect of developed model system including three types of matrices including d‐limonene microcapsule in water, flavored rock candy in water and flavored rock candy in water and citric acid was investigated at three temperatures (10, 45, and 75°C) on the perception and release of d‐limonene by developed time–intensity (TI) sensory analysis and headspace gas chromatography, respectively.

Time–intensity profiles indicate that the presence of sucrose in the matrix of flavored rock candy in water increases the release of d‐limonene due to the salting out effect of sucrose. On the other hand, the addition of citric acid (pH = 3) to the matrix resulted in a significant increase (*p* < .05) in d‐limonene perception which can be attributed to the change in equilibrium between citric acid and its dissociated form since, at pH = 3, there is less of the dissociated form of citric acid in the model system. Results suggested that serving temperatures influence the release and perception of d‐limonene, as physical‐chemical properties of this tastant is impressively changed by increasing the temperature in three spot degrees as 10, 45, and 75°C.

The investigation of d‐limonene release by headspace gas chromatography provided evidence on the data obtained by time–intensity sensory analysis. Results showed that the developed model system and time–intensity method could efficiently describe flavor release from rock candy and could benefit industry who are using encapsulated flavors in their products.

## CONFLICT OF INTEREST

None declared.

## ETHICAL APPROVAL

This study was approved by Department of Food Science and Technology, Faculty of Agriculture, Ferdowsi University of Mashhad.
